# Antibacterial Effects of Quinazolin-4(3*H*)-One Functionalized-Conjugated Silver Nanoparticles

**DOI:** 10.3390/antibiotics8040179

**Published:** 2019-10-09

**Authors:** Abdulkader Masri, Ayaz Anwar, Naveed Ahmed Khan, Muhammad Saquib Shahbaz, Khalid Mohammed Khan, Syed Shahabuddin, Ruqaiyyah Siddiqui

**Affiliations:** 1 Department of Biological Sciences, School of Science and Technology, Sunway University, Selangor 47500, Malaysia; ayaz@hotmail.com (A.A.); naveed5438@gmail.com (N.A.K.); ruqaiyyahs@sunway.edu.my (R.S.); 2 HEJ Research Institute of Chemistry, International Center for Chemical and Biological Sciences, University of Karachi, Karachi City, Sindh 75270, Pakistan; saquib.shah48@yahoo.com (M.S.S.); khalid.khan@iccs.edu (K.M.K.); 3 Department of Clinical Pharmacy, Institute for Research and Medical Consultations (IRMC), Imam Abdulrahman Bin Faisal University, P.O. Dammam Box 31441, Saudi Arabia; 4 Research Center for Nano-Materials and Energy Technology (RCNMET), School of Science and Technology, Sunway University, Selangor Darul Ehsan 47500, Malaysia; syeds@sunway.edu.my

**Keywords:** Quinazolinone, characterization, antibacterial, silver nanoparticles

## Abstract

Infections due to multi-drug resistant bacteria are on the rise and there is an urgent need to develop new antibacterials. In this regard, a series of six functionally diverse new quinazolinone compounds were synthesized by a facile one-pot reaction of benzoic acid derivatives, trimethoxymethane and aniline derivatives. Three compounds of 3-aryl-8-methylquinazolin-4(3H)-one, and 3-aryl-6,7-dimethoxyquinazolin4(3H)-one were prepared and tested against multi-drug resistant bacteria. Furthermore, we determined whether conjugation with silver nanoparticles improved the antibacterial efficacy of these quinazolinone derivatives. The newly synthesized compounds were characterized by ultraviolet visible spectrophotometry (UV-vis), Zetasizer analysis, Fourier transform infrared spectroscopic methods (FT-IR), and scanning electron microscopy (SEM). Using bactericidal evaluation, effects were determined against selected Gram-negative and Gram-positive bacteria. Furthermore, cytotoxicity of nanoconjugates on human cells were determined. The UV-vis spectrum of silver nanoparticles conjugated quinazolinone displayed surface plasmon resonance band in the range of 400–470 nm, and the size of nanoparticles was detected to be in the range of 100–250 nm by dynamic light scattering (DLS). FT-IR study confirmed the stabilization of silver nanoparticles by the presence of diverse functional arayl on each compound. SEM further revealed the construction of spherical nanoparticles. Among the quinazolinone derivative tested, two compounds (QNZ 4, QNZ 6) conjugated with silver nanoparticles showed enhanced antibacterial activity against *Escherichia coli* K1, *Streptococcus pyogenes*, *Klebsiella pneumoniae*, *B. cereus* and *P. aeruginosa* as compared to the compounds.

## 1. Introduction

Multidrug resistance bacteria (MDR) are on the rise and several pathogenic bacteria have gained resistance to at least one class of usable antibiotics [1,2]. The expression quinolone in general recognition belongs to a set of topoisomerase enzyme inhibitors in bacteria that contains quinazolines, naphthyridones, quinolones, and different associated agents [3]. Quinazolinediones represent a group of antibacterial materials, structurally linked to fluoroquinolones [1]. The wide range of chemotherapeutic efficiency have rendered interest for quinolone [4]. Quinolones act by bacterial chromosome fragmentation, and are generally termed fluoroquinolones because of the inclusion of the fluorine [5]. However, the present quinolones undergo from obstacles such as partial activity against a number of medically important Gram positive bacteria [4]. Quinazolinone (QNZ), which belongs to the nitrogen-containing heterocyclic compounds, is a constituent for roughly 200 natural alkaloids secluded from a number of families from microorganisms, plants, and animals [6,7]. By connecting numerous effective groups to the quinazoline moiety, these compounds have been studied due to their biopharmaceutical activities, in particular anticancer, diuretic, anti-inflammatory, anticonvulsant and antihypertensive qualities [8,9]. Over the last decades, many studies related to quinazolinone substituents were performed; these reports involved a range methods of synthesis, characterizations, biomedical therapies, QNZ loading into diverse forms of nanoparticles, and integration of nanoparticles in QNZ production [10]. For example, in multicomponent reaction, the magnetic nanoparticles were used to support the catalyst factor and improve their reactivity and reusability and prevent catalyst separation from the reaction media [11].

The chemical structures of QNZ derivatives, types of nanoparticles (NPs), and ways of QNZ derivatives synthesis play important role in QNZ release behavior, antimicrobial activity and cytotoxicity effects. Generally, the drug release behavior depends on a number of factors that included particle size, and surface properties [12]. For QNZ nanoconjugates, QNZ release rate in acidic pH is greater than that at basic medium, and the morphology of the nanosphere is changed according to the functional group of quinazolinone to nanorod [12,13]. QNZs are an important class of compounds with known antibacterial implications, however these highly functionalized synthetic QNZ were conjugated with nanoparticles for the first time. Moreover, these nanoconjugates have also been tested for antibacterial activity in for the first time.

To explore the effects of loading QNZ derivatives on silver nanoparticles (AgNPs), in enhancing the biological activity, here we synthesized AgNPs as carriers, to provide efficient loading of QNZ derivatives. The nanomaterials were rigorously characterized by using spectroscopic systems. These nanoparticles were tested for the first time against several MDR bacteria including: *Escherichia coli* K1 (*E. coli* K1), Methicillin-resistant *Staphylococcus aureus* (MRSA), *Streptococcus pyogenes* (*S. pyogenes*), *Klebsiella pneumoniae* (*K. pneumoniae*), *Bacillus cereus* (*B. cereus*), and *Pseudomonas aeruginosa* (*P. aeruginosa*).

## 2. Results

### 2.1. Quinazolinone Derivatives Did Not Show Bactericidal Effects

Initial experiments to determine antibacterial effects of the six novel QNZ derivatives [1,2,3,4,5,6] against *E. coli* K1 were performed at 100 µg/mL. Methanol and 100 µg/mL gentamicin were used as negative and positive controls respectively. The results revealed that none of the QNZs tested had any effect on the bacteria. There were no bacteria observed when incubated with gentamicin. However, when QNZ 2, QNZ 5 were incubated with *E. coli* K1, the percentage of bacteria viability was 97%, this was similar to when incubated with QNZ 1, QNZ 3, QNZ 6 and the percentage were (89%, 88%, 84%) respectively.

### 2.2. Characterization of QNZ-AgNPs

**Figure 1:**
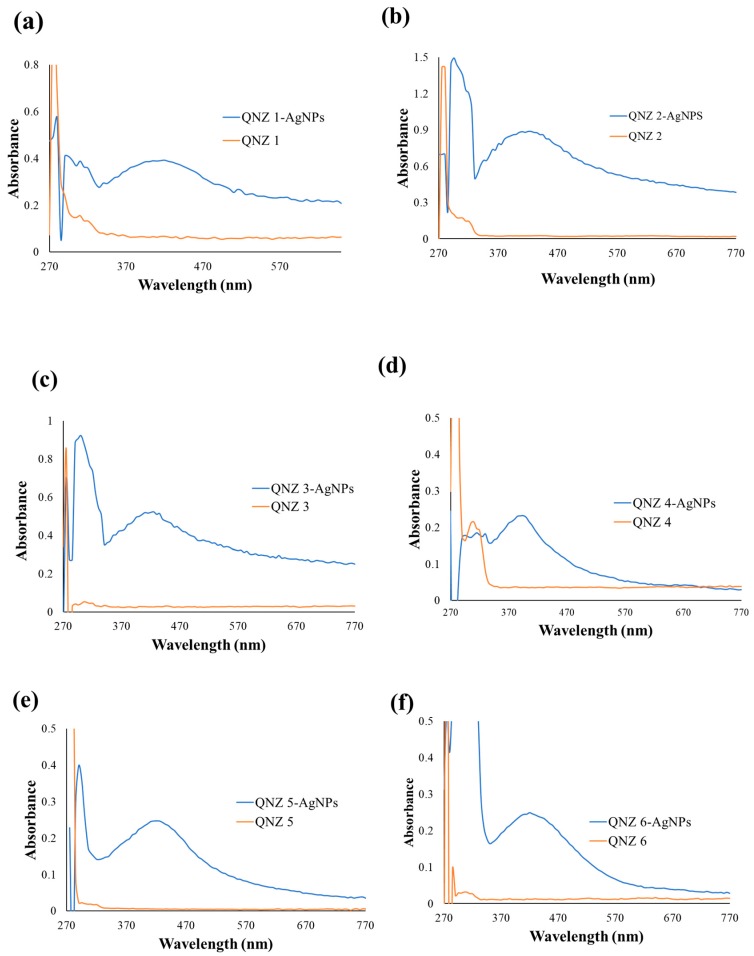
UV-vis spectrum of Quinazolinone (QNZ) 1, QNZ 1-AgNPs (**a**). QNZ 2, QNZ 2-AgNPs (**b**). QNZ 3, QNZ 3-AgNPs (**c**). QNZ 4, QNZ 4-AgNPs (**d**). QNZ 5, QNZ 5-AgNPs (**e**). QNZ 6, QNZ 6-AgNPs (**f**). The spectrum of each QNZ is represented in red line and in blue line after conjugations with silver, the nanoconjugate showed characteristic surface plasmon resonance band at 450 nm.

The UV-vis spectra of quinazolinone alone [1,2,3,4,5,6] and their silver nanoconjugates were tested. The existence of surface plasmon resonance (SPR) which results from the interaction between free electrons in metal nanoparticles and incident light confirms the formation of nanoparticles and a characteristic SPR band in the range of 400–470 nm was observed (Figure 1).

**Figure 2:**
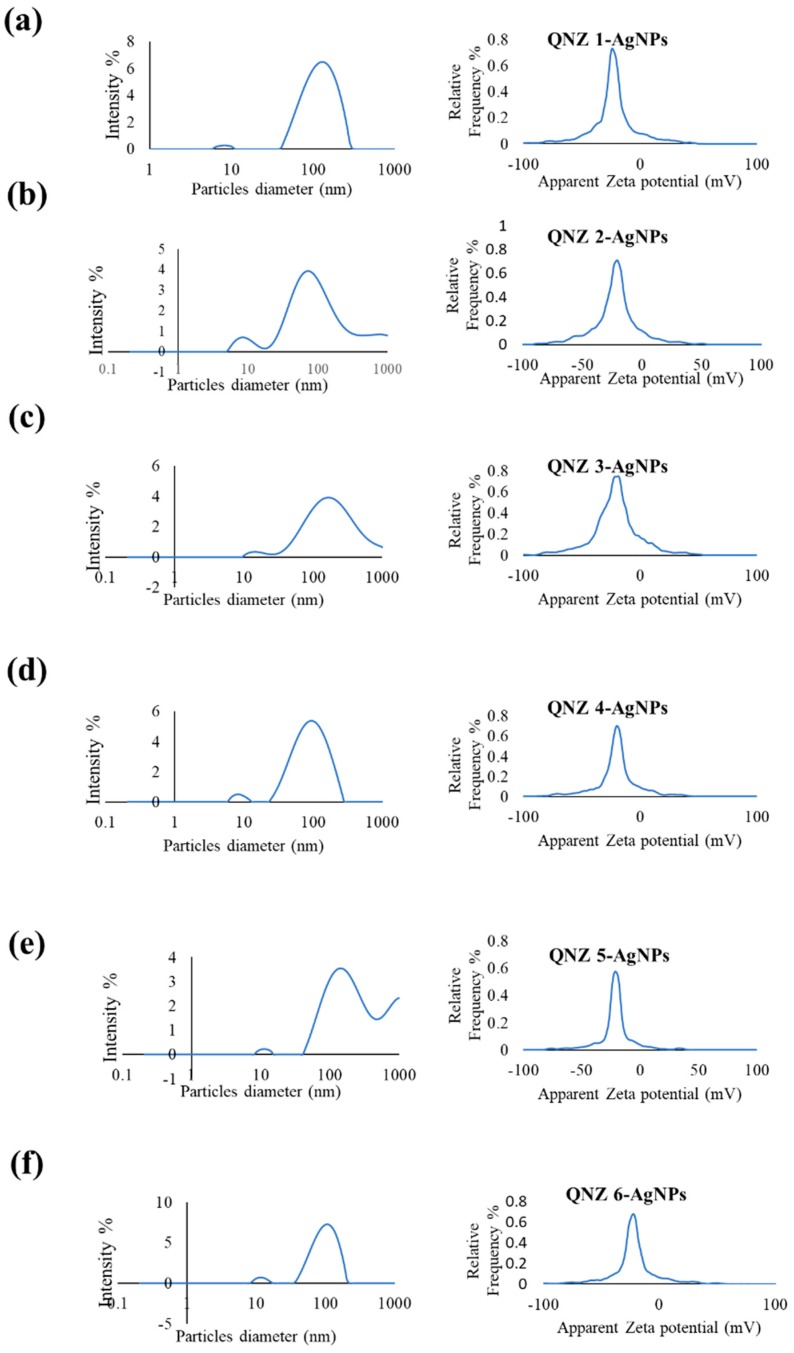
Size distribution histogram and zeta potential plots of QNZ 1-AgNPs (**a**). QNZ 2-AgNPs (**b**). QNZ 3-AgNPs (**c**). QNZ 4-AgNPs (**d**). QNZ 5-AgNPs (**e**). QNZ 6-AgNPs (**f**). which were obtained using Litesizer 500, Anton Paar instrument. The nanoparticles showed diameter in the range of 80–300 nm. Zeta potential plots showed the negative surface charge of nanoconjugate which affects the stability.

The shape and size of nanoparticles is important in the plan of drug delivery systems. A high surface area to volume ratio results from reducing the size of nanoparticles, followed by increased opportunities of interactions between bioactive drug molecules and nanoparticles eventually improving the therapeutic efficiency of the drug [14]. The mean size distribution of QNZ 1-AgNPs, QNZ 2-AgNPs, QNZ 3-AgNPs, QNZ 4-AgNPs, QNZ 5-AgNPs, and QNZ 6-AgNPs were found to be 112, 107, 203, 93, 280, and 99 nm respectively (Figure 2). Large mean size of the QNZ-AgNPs is attributed to uneven scattering of the QNZ moieties over the surface of silver nanoparticles. Zeta potential of QNZ 1-AgNPs, QNZ 2-AgNPs, QNZ 3-AgNPs, QNZ 4-AgNPs, QNZ 5-AgNPs, and QNZ 6-AgNPs were found to be −22.3 ± 0.7, −23.4 ± 0.4, −24.1 ± 0.6, −24.8 ± 0.5, −21.9 ± 0.5, and −25.1 ± 0.6 mV respectively. Zeta potential is a significant factor for the determination of nano-carrier stability. The results show nearly similar zeta potential to all samples due to similar surface chemistry of QNZ-AgNPs (Figure 2a–f).

**Figure 3:**
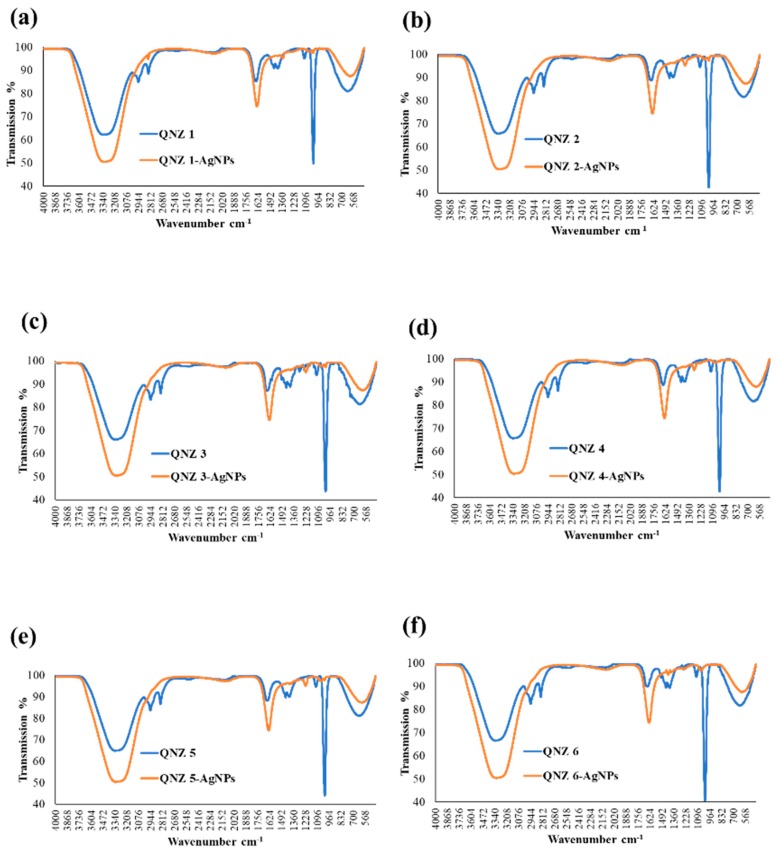
FT-IR spectra of each quinazolinone is compared with their nanoconjugate with AgNPs. QNZ 1, QNZ 1-AgNPs (**a**). QNZ 2, QNZ 2-AgNPs (**b**). QNZ 3, QNZ 3-AgNPs (**c**). QNZ 4, QNZ 4-AgNPs (**d**). QNZ 5, QNZ 5-AgNPs (**e**). QNZ 6, QNZ 6-AgNPs (**f**). The spectrum of each QNZ is represented in blue line and in red line after conjugations with silver. The spectra were obtained by adding 10 µL of the material into FT-IR spectrometer.

**Figure 4:**
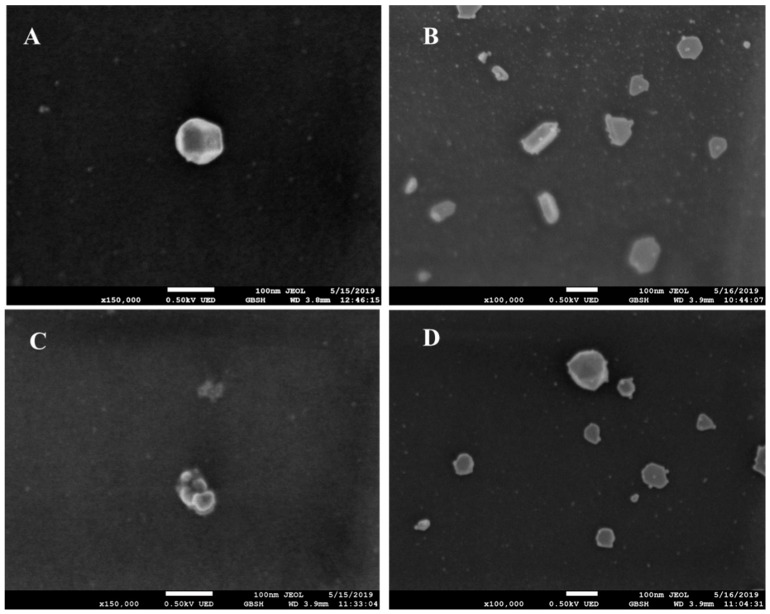
SEM images of AgNPs with magnification 150,000× (**A**), AgNPs/100,000× (**B**), QNZ 6-AgNPs/150,000× (**C**), QNZ 6-AgNPs/100,000× (**D**). Images were recorded on field-emission scanning electron microscope (FE-SEM) (JEOL, 7800F) instrument. Scale bar represents 100 nm.

FT-IR spectrum nanoconjugates exhibited characteristic peaks for the functional groups present and the spectrum of each nanoconjugate with their quinazolinone derivative are presented in Figure 3. FT-IR spectra of QNZ [1,2,3,4,5,6] show multiple peaks at 3440, 1681, 1402, and 1107 cm^−1^, these peaks are related to N–H, C=O, C=N, C–N stretching vibration, respectively. Upon loading quinazolinones with silver nanoparticles, all bands are observed with slight shifting and broadening. The peaks of QNZ were shifted to lower wavenumber 3437, 1648, 1347, and 1053 cm^−1^ in QNZ-AgNPs showing metal and ligand interaction. These data suggest that QNZ was successfully loaded onto AgNPs by physical interaction and incorporation confirmed the chemical stability. From a typical SEM image of a thin coating made from quinazolinone (6) solution, it was observed that the particles are spherical in shape, have an average size of 100 nm and are consistently dispersed (Figure 4).

### 2.3. QNZ 4 and QNZ 6 Conjugated Silver Nanoparticles Exhibited Bactericidal Effects

**Figure 5:**
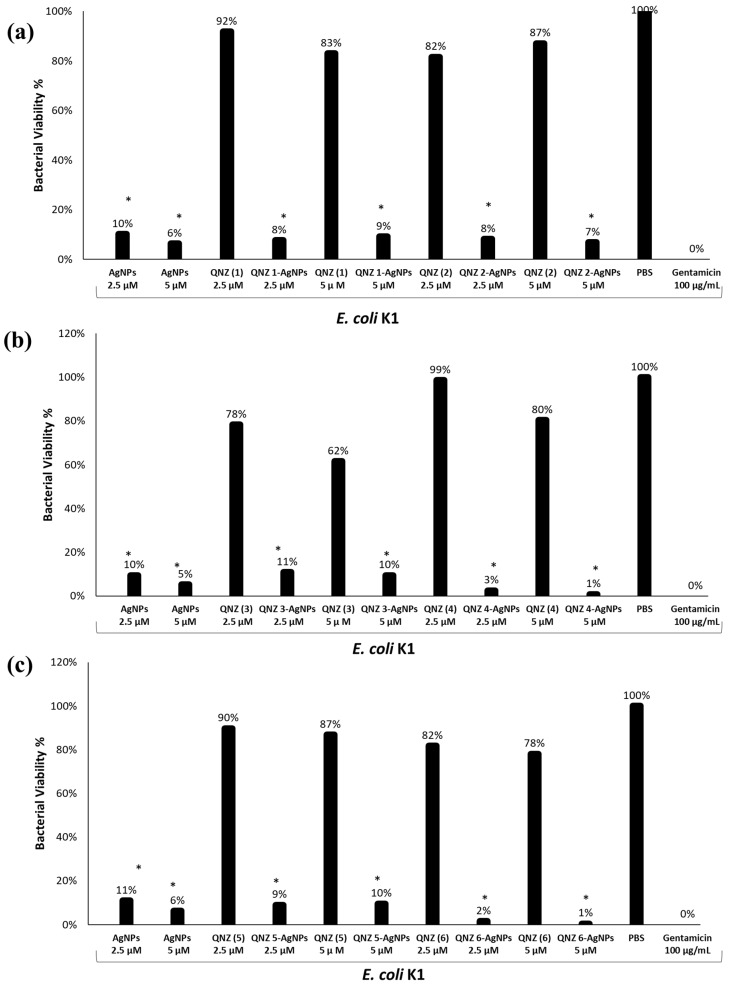
Bactericidal assay against *E. coli* K1(**a**–**c**). The viability of bacteria was determined after assay as described in the materials and methods section. Briefly, 10^6^ colony forming units (C.F.U.) were incubated with AgNPs alone, QNZ alone, and QNZ-AgNPs and negative and positive controls at 2.5 and 5 µM at 37 °C for 2 h. The next day, the cells were counted. Note all nanoconjugates showed bactericidal effects comparing with *E. coli* K1 alone but QNZ 4-AgNPs and QNZ 6-AgNPs had lower percentage of availability compared with AgNPs. The results are presented as the mean ± standard error of various experiments performed in duplicate. * indicates *p* < 0.05 using two-sample *t* test and two-tailed distribution.

**Figure 6:**
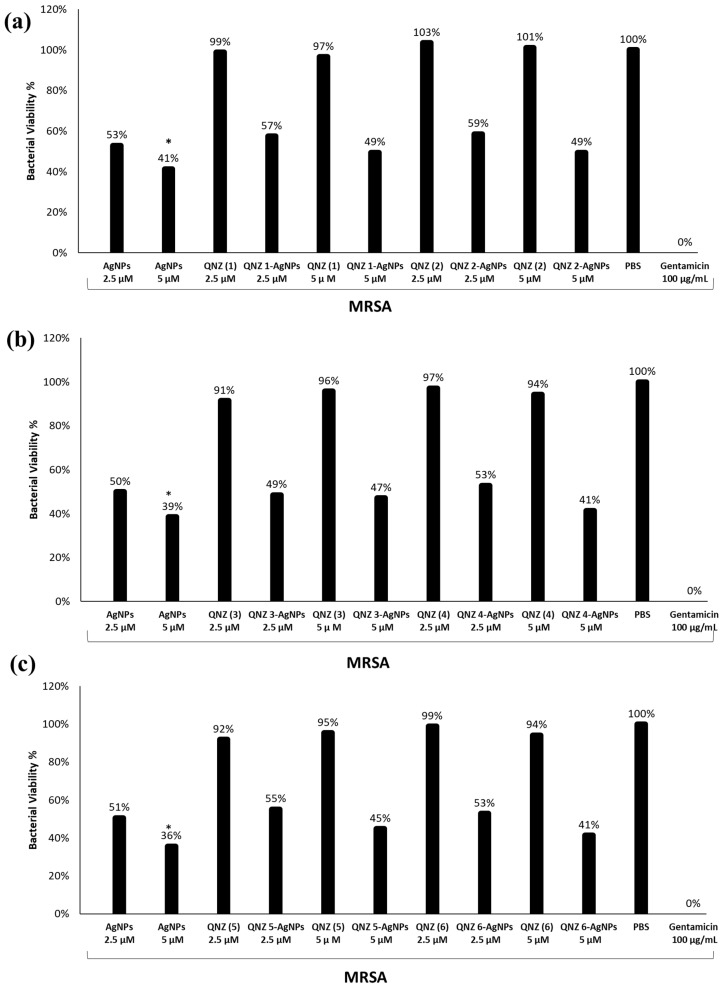
Bactericidal assay against MRSA (**a**–**c**). Note that all nanoconjugates did not exhibit bactericidal effects against MRSA. The results are presented as the mean ± standard error of various experiments performed in duplicate. * indicates *p* < 0.05 using two-sample *t* test and two-tailed distribution.

**Figure 7:**
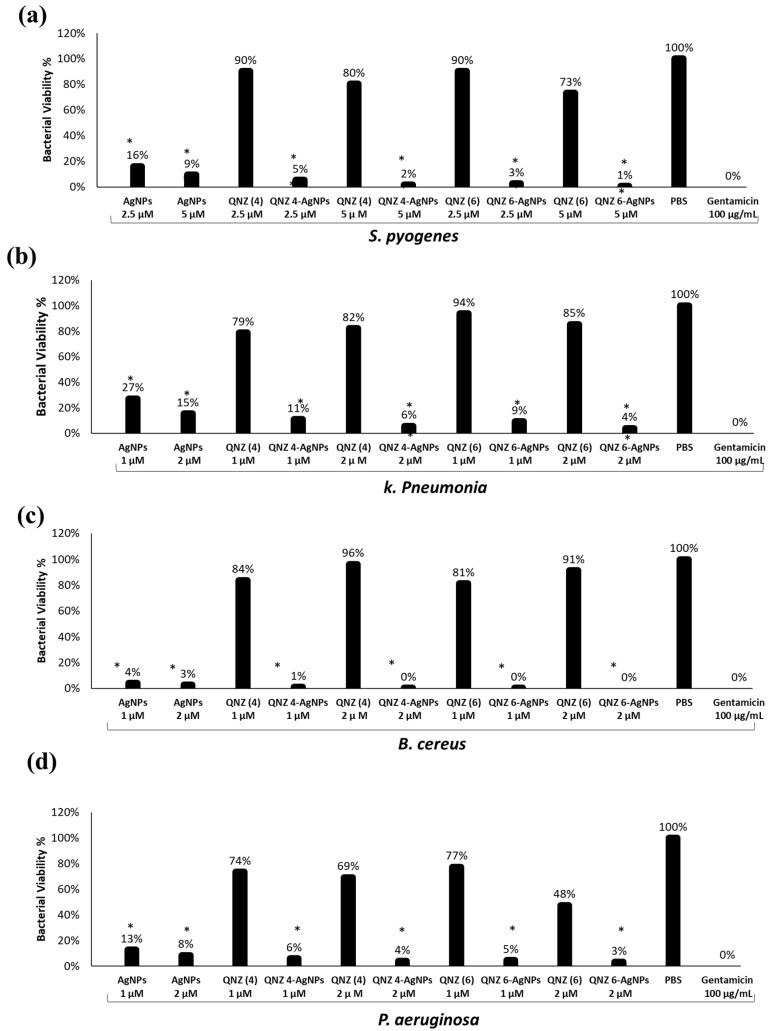
Antibacterial activities of quinazolinone nanoparticles against multi-drug resistant bacteria. (**a**) Bactericidal effects of QNZ 4-AgNPs and QNZ 6-AgNPs at (2.5, 5) µM against S. pyogenes. (**b**) Bactericidal effects of QNZ 4-AgNPs and QNZ 6-AgNPs at (1, 2) µM against K. pneumonia. (**c**) Bactericidal effects of QNZ 4-AgNPs and QNZ 6-AgNPs at (1, 2) µM against *B*. cereus. (**d**) Bactericidal effects of QNZ 4-AgNPs and QNZ 6-AgNPs at (1, 2) µM against *P*. aeruginosa. Notably, nanoparticles showed significant effects at both concentration against both *S*. pyogenes and *K*. pneumonia comparing with bacteria alone and lower bacterial viability as compare to AgNPs alone. Also, the nanoconjugates displayed minimal bactericidal effects against *B*. cereus and *P*. aeruginosa compared with AgNPs. The results are presented as the mean ± standard error of various experiments performed in duplicate. * indicates *p* < 0.05 using two-sample *t* test and two-tailed distribution.

The in vitro antibacterial activity of AgNPs loaded QNZ derivatives was evaluated against six selected bacteria. Figure 5 and Figure 6 represents the bactericidal effects of six quinazolinone [1,2,3,4,5,6] conjugated AgNPs tested at 2.5 and 5 µM against *E. coli* K1 and MRSA. Among the tested compounds, QNZ 4-AgNPs and QNZ 6-AgNPs showed potent bactericidal activity at both concentration against *E. coli* K1 (Figure 5), but all samples did not exhibit significant effects against MRSA compared with AgNPs alone (Figure 6). Both QNZ 4, QNZ 6 conjugated with AgNPs exhibited bactericidal effects against Gram positive *S. pyogenes* (Figure 7a). Also, the same compounds at 1 and 2 µM showed activity against *K. pneumonia* compared with bare AgNPs (Figure 7b). However, both derivatives showed minimal effects against *B. cereus* and *P. aeruginosa* comparing with AgNPs alone (Figure 7c,d). Overall the findings showed that the bactericidal effects of QNZ were significantly increased after conjugation with AgNPs (Table 1). 

### 2.4. Effect of Incubation Time

**Figure 8:**
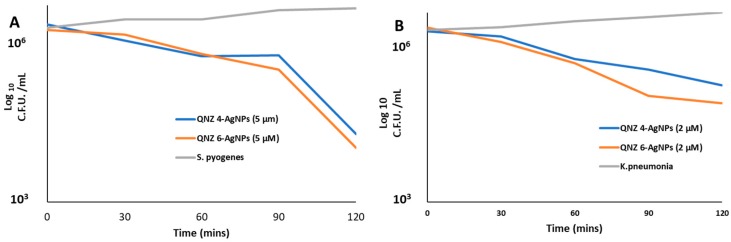
Antimicrobial effect of QNZ nanoparticles against *S. Pyogenes* (**A**) and *K. Pneumonia* (**B**). Both concentrations of nanoparticles were applied to approx. 10^8^ colony forming units (C.F.U.) per mL of bacteria. At different times after the treatment, viable cell counts were measured by culturing bacterial colonies on agar plates. Each of the CFU/mL value represents the mean of three replicates.

The observed antimicrobial effect was further investigated over a set time trial. Live cells were measured by the colony forming units on agar. At a same concentration of 5 µM QNZ 4-AgNPs and QNZ 6- AgNPs, *S. pyogenes* was almost completely killed within 2 h to reach 2 × 10^4^ and 10^4^ respectively (Figure 8A). At 2 µM, *K. pneumonia* was gradually reduced after 120 min to reach 1.4 × 10^5^ and 3.6 × 10^5^ respectively (Figure 8B). This demonstrates that QNZ nanoparticles are effective at killing both bacteria at low concentrations in short periods of time.

### 2.5. QNZs Displayed Minimum Cytotoxicity to Human Cells and Abolished Bacterial Mediated Cytotoxicity to Human Cells

**Figure 9:**
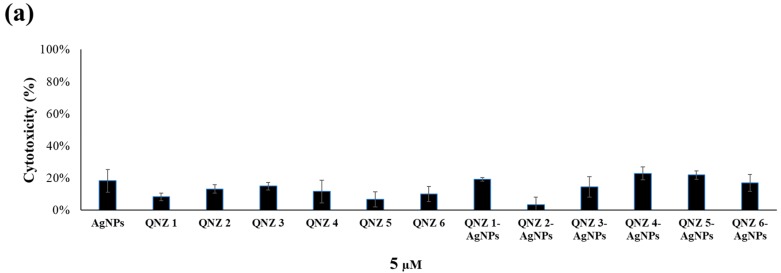

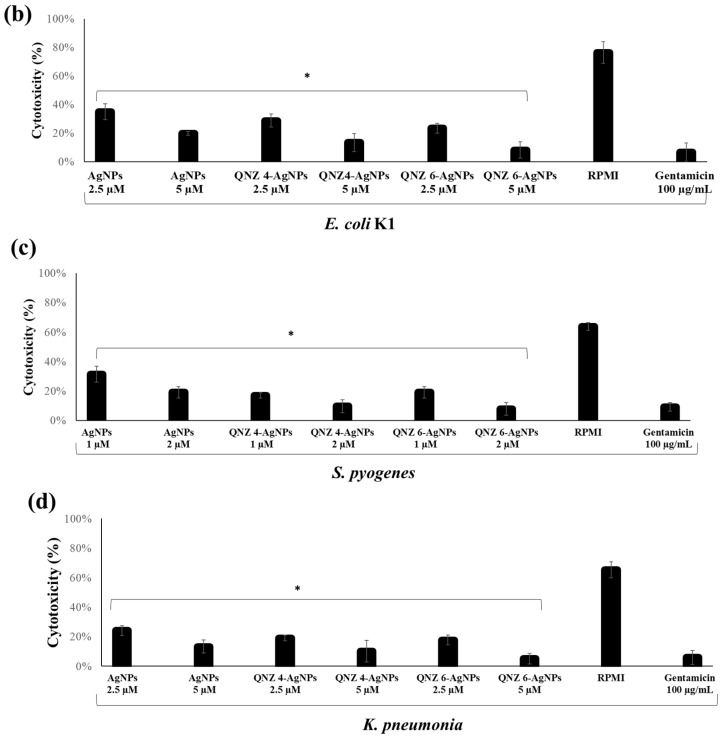
(**a**) The quinazolinone derivatives and their silver nanoparticles did not exhibit cytotoxicity against Human keratinocyte cells at 5 µM. These nanoparticles and the respective controls were incubated with HaCaT cells monolayer for 24 h at 37 °C in a 5% CO_2_ incubator. Following this incubation, cell-free supernatant was collected, and cytotoxicity was determined using Lactate dehydrogenase (LDH) assay kit (Roche). The negative control values for cytotoxicity assays were obtained by incubating HaCaT cells with RPMI-1640 alone, and positive control values were obtained by 100% cell death using 0.1% Triton X-100. (**b**,**c**) Pretreatment of 2.5 and 5 µM of QNZ 4-AgNPs and QNZ 6-AgNPs abolished *E. coli* K1 and *S. pyogenes*-mediated host cells cytotoxicity, *E. coli* K1 caused 70% cytotoxicity to HaCaT cells. Upon pretreatment with 5 µM QNZ 6-AgNPs, the host cells cytotoxicity was reduced to 4%. (**d**) Pretreatment of 1 and 2 µM of QNZ 4-AgNPs and QNZ 6-AgNPs abolished *K. pneumonia*-mediated host cells cytotoxicity. The results are presented as the mean ± standard error of various experiments performed in duplicate. * indicates *p* < 0.05 using two-sample *t* test and two-tailed distribution.

All test samples displayed minimum cytotoxic effects when tested against non-cancerous human cells (Figure 9a). Both quinazolinone derivatives alone [1,2,3,4,5,6] and their nanoconjugates produced less than 20% cytotoxicity against Human keratinocyte cells at a concentration of 5 µM. Pretreatment of QNZ 4-AgNPs and QNZ 6-AgNPs with *E. coli* K1 and *S. pyogenes* resulted in decline of their cytopathogenicity against human cells. Figure 9b shows that untreated *E. coli* K1 caused more than 70% cell cytotoxicity against HaCaT cells, while *E. coli* K1 treated with AgNPs caused almost 20% cell death while in conjugation with QNZ 6, and at the same concentration, the toxicity was reduced to less than 5%. Similarly, pretreatment of both conjugations with *K. pneumonia* abolished bacterial mediated cytotoxicity of HaCaT cells (Figure 9d) as compared to untreated *K. pneumonia* which exhibited 64% cytotoxicity.

## 3. Discussion

Nanoparticles are one of the promising alternative approaches in the development of antibacterial chemotherapy [15]. Here we tested novel QNZ- derivatives for potential antibacterial purposes. A structure-activity relationship was studied among the six QNZ derivatives used in this study. The overall results indicate that the variation in activity was observed based on the different substituents present on the QNZ ring and their respective positions on the aryl part. The presence of electron donating methoxy groups on QNZ (compounds 4, 5, 6) core structure gave better activity as compared with methyl group (compounds 1, 2, 3). Within the 3-aryl-6,7-dimethxyquinazolin-4(3H)-ones the substitutions on aryl ring of compounds 4, 5, and 6 had different effects on the antibacterial properties. The addition of electron donor groups such as methylthio (4) and methoxy (6) groups exhibited optimal bactericidal activity when conjugated with silver nanoparticles. The reason for enhanced antibacterial effects of quinazolinones with electron donating groups may be attributed to their ability to stabilize the nanoparticles more efficiently while developing more electrostatic charge on the surface of nanoparticles which may lead to better interaction with bacteria. These results are also in agreement with the dynamic light scattering (DLS) data where QNZ 4-AgNPs and QNZ 6-AgNPs showed lowest zeta potential and smallest size in the series. However, these two materials were not active against all six bacteria tested.

Furthermore, the chemical structure and nature of the substituent is critical to increase the bioactivity of a compound [16]. For instance, the attachment of fluoro group with phenyl ring of the nanoconjugates behaves as an active substrate in growth inhibition of the bacteria and the introduction of p-methoxy phenyl group reduces its antibacterial efficacy [4,5,6,7,8]. The angular isomers of quinazolinone revealed weak inhibitory capacity than the linear composites [6]. Also, due to the resemblance in structure of indole core with naturally compounds, Rohini et al. (2010) reported that they can simply interact with molecules of the biological systems as studies on the indole-substituted quinazolines showed inhibition against the tested microorganism especially Gram negative bacteria [17]. Dahiya et al. (2008) synthesized substituted quinazoline/imidazolyl-salicylic acid conjugated with amino acid which were assayed against eight pathogenic microbes [18]. Two compounds exhibited antibacterial activity against *K. pneumoniae* and *P. aeruginosa* [18]. Furthermore, the phenyl ureas substituted quinazolinone compounds have also shown growth reduction of *E. coli* at 40 μg/mL [19]. In a recent study Divar et al. (2018) compared the 10 QNZ derivatives and the findings displayed that replacement of 2 chlorine atoms on quinazolinone core ring improved the antimicrobial action and substitution of methyl in corporation aniline ring with methoxy residue decreases its antimicrobial activities [16]. It could be supposed that the presence of methyl in corporation to the aniline ring together with chlorine atoms at ortho- and meta- positions of the quinazoline ring enhances the antimicrobial activity against a wide range of microbes [20]. Rana and Desai (2013) demonstrated that compounds having electron donating groups (–SCH3) at the phenyl were found to be more potent and showed inhibition at 40 µg/mL against *E. coli*, while compounds having electron withdrawing groups (–Cl, –CF3) were found to be less potent. We also observed similar effects, nearly in the case of AgNPs conjugation with QNZ 4 and QNZ 6 [19].

It is known that chemical groups at various positions affect activity differently. For example, quinazoline derivatives with substituted amine on 4-position and halogens on 6- position could promote the antimicrobial activities [6]. Previously, it has been shown that substitution of heterocyclic moiety at C6 position showed outstanding activity against MDR bacteria [17] and this corresponds with the existence of methoxy group at C6 in compounds QNZ 4, QNZ 6 in our study. Furthermore, the activity of synthetic compounds could also have specific effects against a particular bacteria only [16], which is consistent with our findings. Moreover, the nanocarriers QNZ 4, QNZ 6 did not exhibit cytotoxicity against human cells at same concentration, however, they reduced bacterial mediated host cell cytotoxicity, indicating that they would be good candidates as antibacterial agents.

Previous studies illustrate the antimicrobial efficacy of nanoparticles loaded with QNZ derivative [12,13,14,15,16]. For example, polypyrolol conjugated chitosan loaded with QNZ revealed higher activity against both Gram negative and Gram positive bacteria than chitosan and polypyrolol alone and this improved activity is associated with effecting both the protonated amino group of QNZ with amino group of chitosan and polypyrolol [12,13]. Furthermore, the synergistic effects of nanocomposite PPy AgCl that combine the properties of silver chloride make the compound an ideal carrier for 3-amino-2 phenyl-4(3H)-quinazolinone (I) and has antibacterial activity against *E. coli* due to the presence of AgCl nanoparticles. These results were attributed to the presence of positive charge in the nanoconjugate and negative charge of bacteria cell wall [21]. 

It has been well documented that silver nanoparticles have many opportunities to interrupt biochemical progression and inhibit bacterial multiplication. The possible mechanism could be associated with interaction between bacteria and nanoparticles [22,23]. The mechanism of these is thought to lead to interactions increasing amounts of reactive oxygen species primarily hydroxyl radical and less stable singlet oxygen, followed by deposition of the nanoparticle on the surface of bacteria or accumulation in preplasmic region which causes destruction of cellular function and disorder of membranes [24,25]. The structural resemblance between quinazolinones and fluoroquinolone suggest a similar mode of action that is inhibition of Topoisomerase I (DNA gyrase) which is a crucial enzyme required for DNA multiplication in bacteria during supercoiling, replication, and separation of chromosomal DNA. Moreover, chemical modification of structure of QNZ derivatives enhance their antibacterial activity. Further studies to study the mechanism of action for QNZ 4 and QNZ 6 conjugated with AgNPs need to be performed.

## 4. Materials and Methods 

Various QNZ derivatives (Table 2), were synthesized by functionally different compounds of each 3-aryl-8-methylquinazolin-4(3H)-ones and 3-aryl-6,7-dimethxyquinazolin-4(3H)-ones. These derivatives were analyzed through proton nuclear magnetic resonance (1HNMR), electron ionization mass spectrometry (EI-MS), and elemental analysis. All reagents and chemicals were obtained from Sigma-Aldrich unless stated otherwise. Silver nitrate salt was the initial material for the making of silver nanoparticles. The reducing agent was Sodium borohydride (NaBH4) >95.0% (TCI, Tokyo, Japan).

### 4.1. Synthesis of Quinazolinones

**Scheme 1:**
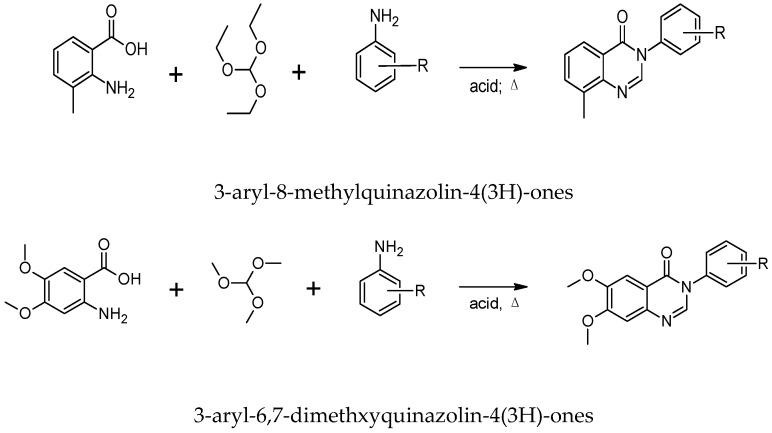
The chemical synthesis of six quinazolinone derivatives.

A library of six analogs of Quinazolin-4(3H)-ones were synthesized via one pot reaction of benzoic acid derivatives (1 mmol) with triethoxymethane (3 mmol) and different substituted anilines (1 mmol) in acidic medium using acetic acid (Scheme 1). The reaction combination was boiled and kept on reflux, while the reaction progress was examined with thin layer chromatography. After the complete absence of starting materials, the reaction mixture was poured in water, precipitate formed were filtered, thoroughly washed with ultrapure water and dried under vacuum and crystallized.

### 4.2. Characterization of Quinazolinones 

QNZ derivatives were elucidated by 1H-NMR and EI-MS. Elemental analyses and high resolution EI-MS data of all compounds were found to be in consistent scope (Table 3). The chromatographic and spectroscopic analyses also confirmed high purity (>95%) of all compounds.

### 4.3. Bacterial Cultures

Cultures of three Gram negative bacteria were utilized, comprising: *E. coli* K1 which is a neuropathogenic, and was derived from a cerebrospinal fluid of meningitis patient, strain E44; O18:K1:H7, Malaysian Type Culture Collection (MTCC) 710859, *K. pneumoniae*; American Type Culture Collection (ATCC 13883), and *P. aeruginosa* (ATCC 10145). Three Gram positive bacteria were also utilized, comprising: methicillin-resistant *S. aureus* (MRSA) strain, extracted from patient blood cultures (MTCC 381123), (Luton & Dunstable Hospital NHS Foundation Trust, England, UK), *S. pyogenes* (ATCC 49399), and *B. cereus* (MTCC 131621). Bacterial cultures were restored by sub-culturing every two weeks on nutrient agar plates and were kept at 4 °C [26].

### 4.4. Human Keratinocyte (HaCaT) Cells Culture

Human keratinocyte cells were cultivated in Roswell Park Memorial Institute (RPMI)-1640 augmented with glutamine (2 mM), fetal bovine serum (10%), Nu-serum (10%), penicillin (100 units/mL), streptomycin (100 μg/mL), pyruvate(1 mM), vitamins, and non-essential amino acids (NEAA) to obtain identical monolayers of HaCaT cells in 75 cm^−2^ culture flasks as described previously [27]. Briefly media was removed, and cells were exposed to 2 mL trypsin. After incubation for 15 min, the cell suspension was centrifuged for 5 min at 2500× *g*, and the cell pellet was resuspended in new supplemented media. 200 μL of this cell suspension was seeded in each well of a 96-well plate and the plate was incubated at 37 °C in a 5% CO_2_ incubator with 95% humidity for 24 h until formation of uniform monolayer of HaCaT cells. These were used for cytotoxicity and bacteria mediated-host cells cytotoxicity assays [28].

### 4.5. Synthesis of AgNPs Coated with Quinazolinione

For the synthesis of QNZ derivatives conjugated with silver nanoparticles (QNZ-AgNPs), 3 mL (0.1 mM) QNZ derivatives aqueous solution was reacted with 3 mL (0.1 mM) aqueous solution of silver nitrate, and the reaction blend was magnetically stirred for 10 min. Following this, 20 μL of 5 mM freshly prepared (NaBH4) aqueous solution was added in above stirring reaction mixture. The color of solution turned yellow-brown from transparent on addition of the reducing agent indicating the reduction of silver ions and formation of QNZ-AgNPs [29]. All QNZ derivatives (1,2,3,4,5,6) were conjugated with AgNPs and same process was replicated by adjusting diverse volume ratio (*v*/*v*) of QNZ and silver nitrate solution.

### 4.6. Characterization of Nanoparticles

After successful formation of nanoconjugates, QNZ-AgNPs and QNZ were examined as previously described, by ultraviolet-visible spectrophotometry (UV-visible) and the absorption spectra were measured using Thermo scientific (Evolution 201), Fourier transformation infrared (FT-IR) with frequency wavenumber from 4000 cm^−1^ to 400 cm^−1^, size and zeta potential analysis [15]. Analytical scanning electron micrographs were achieved by means of a JEOL field emission scanning electron microscope (FESEM) with surface observation using gentle beam super high resolution (JSM 7800F, Japan, Tokyo) [14]. Samples were prepared by drying a 10 µL of each of nanoparticles. FT-IR analysis of nanoparticles in comparison to QNZ derivatives were carried out and the spectra were recorded in the frequency range of 400–400 cm^−1^ using FT-IR spectrometer (PerkinElmer, San Francisco, USA), The size of nanoparticles was investigated through particle analyzer (Litesizer 500, Anton Paar, USA). Nanoparticles were moved into the transparent plastic cuvette and read by using distilled water as dispersant agent at 25 °C. Zeta potential was determined. SEM was performed as described previously [30].

### 4.7. Bactericidal Assay

Antibacterial capacity of AgNPs, QNZ and QNZ-AgNPs was detected by performing bactericidal assays [14]. In brief, by using a spectrophotometer the cultures of bacterial were optimized to optical density (OD) of 0.22 at 595 nm (OD 595 = 0.22) which corresponds to 10^8^ colony forming units C.F.U. per mL. An inoculum of 10 μL (10^6^ C.F.U.) of the above bacteria culture was incubated with different concentrations of QNZ derivatives conjugated AgNPs in 1.5 mL Eppendorf tubes at 37 °C for 2 h. For positive controls untreated bacterial culture were incubated with100 μg/mL of gentamicin, while bacteria incubated with phosphate buffer saline (PBS) were used as negative control. QNZ derivatives alone were used as controls. Next, bacteria were serially diluted (ten-fold) and 10 µL of each dilution was plated on agar plates. These plates were incubated at 37 °C overnight, followed by viable bacterial C.F.U. were counted [31].

### 4.8. Time-Kill Kinetics

The antimicrobial effects of QNZ nanoparticles were studied by exposing 108 CFU/mL of S. Pyogenes and K. Pneumonia to 5 and 2 µM of QNZ 4-AgNPs and QNZ 6-AgNPs respectively. The samples were incubated at 37 °C for a total of 2 h. At specific time intervals (0, 30, 60, 90, and 120 min) of incubation, 10 µL of each bacterial suspension was serially diluted and spread on nutrient agar plates and incubated at 37 °C for 24 h. Colonies were counted and represented as log10 CFU/mL of each experiment that was repeated thrice [32].

### 4.9. Cytotoxicity Assay

To estimate the cytotoxic properties of these nanoparticles on human cell, lactate dehydrogenase (LDH) cytotoxicity assay was performed as described previously [28]. Briefly, 5 µM concentrations of QNZ 1-AgNPs, QNZ 2-AgNPs, QNZ 3-AgNPs, QNZ 4-AgNPs QNZ 5-AgNPs and QNZ 6-AgNPs and respective controls were incubated with uniform monolayer of HaCaT cells in a 96 multi well plate, and the cells were incubated for 24 h at 37 °C in a 5% CO_2_ incubator. After 24 h, supernatants were collected from each well and cytotoxicity was elucidated by measuring LDH release by using LDH kit. Untreated cells were considered as negative control, whereas cells incubated with 0.1% Triton X-100 for 20 min gave maximum LDH release as a result of cell lysis which was taken as a positive control. The percentage cell cytotoxicity was calculated as follows: % cell cytotoxicity = (sample absorbance negative control absorbance)/(positive control absorbance negative control absorbance) × 100. The results are representatives of several experiments performed in duplicate and presented as the mean ± standard error.

### 4.10. Bacteria Mediated Host Cell Cytotoxicity

Cytopathogenicity assays were performed as described previously [30,31]. Briefly, *E. coli* K1, *S*. pyogenes and *K. pneumoniae* were incubated with various concentrations of QNZ compounds and their nanoconjugates for 2 h at 37 °C. Next, all test samples were incubated with confluent HaCaT monolayers in RPMI-1640. Plates were incubated at 37 °C for 24 h in a 5% CO_2_ incubator and observed for cytotoxic effects. At the end of this incubation period, supernatants were collected and cytopathogenicity was detected by measuring LDH release. Control values were obtained from host cells incubated in RPMI-1640 medium alone. Total LDH release was determined from HaCaT cells treated with 1% Triton X-100 for 30 min at 37 °C. The basis of this assay is that cell supernatant containing LDH catalyzes the conversion of lactate to pyruvate, generating reduced form of nicotinamide adenine dinucleotide (NADH) and H+. In the second step, the catalyst (diaphorase, solution from kit) transfers H and H+ from NADH and H^+^ to the tetrazolium salt p-iodo-nitrotetrazolium violet (INT), which is reduced to formazon (dye), and absorbance is read at 490 nm.

## 5. Conclusions

After successful conjugation of these derivatives with silver nanoparticles, the results demonstrated potent enhancement of antibacterial efficacy for two nanoconjugates, QNZ 4-AgNPs and QNZ 6-AgNPs. Nonetheless, all quinazolinone derivatives including QNZ 4 and QNZ 6 did not show bactericidal effects against the six tested bacteria in this study. The precise mode of action of these nanoparticles is not exactly comprehended and it is the issue of future studies in company with testing their potential in vivo.

## Figures and Tables

**Table 1:** Differences in bacterial viability percentages between QNZ nanoconjugate and silver nanoparticles alone.

	Nanoparticles					
**Bacterial Viability %**	**AgNPs**		**QNZ 4-AgNPs**		**QNZ 6-AgNPs**	
	2.5 µM	5 µM	2.5 µM	5 µM	2.5 µM	5 µM
***E. coli* K1**	11%	5%	3%	1%	2%	1%
***S. pyogenes***	16%	9%	5%	2%	3%	1%
**MRSA**	No activity					
	1 µM	2 µM	1 µM	2 µM	1 µM	2 µM
***K. pneumonia***	27%	15%	11%	6%	9%	4%
***B. cereus***	4%	3%	1%	0%	0%	0%
***P. aeruginosa***	13%	8%	6%	4%	5%	3%

**Table 2:** The chemical definitions of six quinazolinone derivatives.

Code	Structure	International Union of Pure and Applied Chemistry (IUPAC) Name	Molecular Formula	Molecular Weight
**QNZ-1**	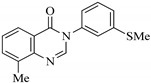	8-methyl-3-(3-(methylthio)phenyl)quinazolin-4(3H)-one	C_16_H_14_N_2_OS	282.36
**QNZ-2**	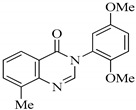	3-(2,5-dimethoxyphenyl)-8-methylquinazolin-4(3H)-one	C_17_H_16_N_2_O_3_	296.32
**QNZ-3**	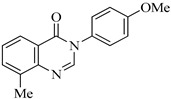	3-(4-methoxyphenyl)-8-methylquinazolin-4(3H)-one	C_16_H_14_N_2_O_2_	266.29
**QNZ-4**	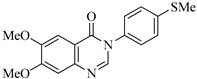	6,7-dimethoxy-3-(4-(methylthio)phenyl)quinazolin-4(3H)-one	C_17_H_16_N_2_O_3_S	328.39
**QNZ-5**	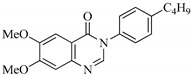	3-(4-butylphenyl)-6,7-dimethoxyquinazolin-4(3H)-one	C_20_H_22_N_2_O_3_	338.40
**QNZ-6**	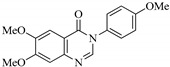	6,7-dimethoxy-3-(4-methoxyphenyl)quinazolin-4(3H)-one	C_17_H_16_N_2_O_4_	312.32

**Table 3:** H-NMR and EI-MS chemical characterization results.

Code	IUPAC Name	Chemical Characterization
**QNZ-1**	8-methyl-3-(3-(methylthio)phenyl)quinazolin-4(3H)-one	Yield: 65%; Rf: 0.76 (ethyl acetate/hexane, 3:7); 1H-NMR: (400 MHz, DMSO-d6): δH 8.36 (s, 1H, H-2), 8.04 (d, J5,6 = 8.0 Hz, 1H, H-5), 7.75 (d, J7,6 = 7.2 Hz, 1H, H-7), 7.50 (t, J6(5,7) = 8.0 Hz, 1H, H-6), 7.49 (t, J5′(6′,4′) = 7.6 Hz, 1H, H-5′), 7.43 (s, 1H, H-2′), 7.39 (d, J6′,5′ = 8.0 Hz, 1H, H-6′); 7.30 (d, J4′,5′ = 7.6 Hz, 1H, H-4′), 2.57 (s, 3H, 8-CH3), 2.50 (s, 3H, 3′-SCH3); EI-MS: m/z (rel. abund. %), 282 [M]+ (100), 267 (5), 254 (3), 123 (4), 105 (15); Anal. calcd. for C16H14N2OS: C, 68.06; H, 5.00; N, 9.92; O, 5.67; S, 11.36; found: C, 68.08; H, 5.04; N, 9.95; O, 5.62; S, 11.34.
**QNZ-2**	3-(2,5-dimethoxyphenyl)-8-methylquinazolin-4(3H)-one	Yield: 81%; Rf: 0.52 (ethyl acetate/hexane, 3:7); 1H-NMR: (300 MHz, DMSO-d6): δH 8.20 (s, 1H, H-2), 8.02 (d, J5,6 = 9.0 Hz, 1H, H-5), 7.74 (d, J7,6 = 7.2 Hz, 1H, H-7), 7.48 (t, J6(5,7) = 7.5 Hz, 1H, H-6), 7.19 (d, J3′,4′ = 9.0 Hz, 1H, H-3′), 7.12 (d, J6′,4′ = 3.0 Hz, 1H, H-6′), 7.09 (dd, J4′,3′ = 8.7 Hz, J4′,6′ = 3.0 Hz, 1H, H-4′), 3.74 (s, 3H, 2′-OCH3), 3.70 (s, 3H, 5′-OCH3) 2.57 (s, 3H, 8-CH3); EI-MS: m/z (rel. abund. %), 296 [M]+ (38), 265 (100), 250 (5); Anal. calcd. for C17H16N2O3: C, 68.91; H, 5.44; N, 9.45; O, 16.20; found: C, 68.94; H, 5.42; N, 9.44; O, 16.23.
**QNZ-3**	3-(4-methoxyphenyl)-8-methylquinazolin-4(3H)-one	Yield: 84%; Rf: 0.62 (ethyl acetate/hexane, 3:7); 1H-NMR: (300 MHz, DMSO-d6): δH 8.32 (s, 1H, H-2), 8.03 (d, J5,6 = 7.8 Hz, 1H, H-5), 7.74 (d, J7,6 = 7.2 Hz, 1H, H-7), 7.48 (t, J6(5,7) = 7.8 Hz, 1H, H-6), 7.45 (d, 2H, J2′,3′ = J6′,5′ = 9.0 Hz, H-2′, H-6′), 7.10 (d, 2H, J3′,2′ = J5′,6′ = 9.0 Hz, H-2′, H-6′), 3.82 (s, 3H, 4′-OCH3), 2.57 (s, 3H, 8-CH3); EI-MS: m/z (rel. abund. %), 266 [M]+ (100), 251 (14), 235 (3); Anal. calcd. for C16H14N2O2: C, 72.16; H, 5.30; N, 10.52; O, 12.02; found: C, 72.18; H, 5.33; N, 10.50; O, 12.00.
**QNZ-4**	6,7-dimethoxy-3-(4-(methylthio)phenyl)quinazolin-4(3H)-one	Yield: 45.45%; Rf: 0.69 (ethyl acetate/hexanes, 7:3); 1H-NMR: (400 MHz, DMSO-d6): δH 8.21 (s, 1H, H-2), 7.49 (s, 1H, H-5), 7.43 (d, J2′,3′ = J6′,5′ = 7.6 Hz, 2H, H-2′, H-6′), 7.41 (d, J3′,2′ = J5′,6′ = 7.6 Hz, 2H, H-3′, H-5′), 7.19 (s, 1H, H-8), 3.92 (s, 3H, 6-OCH3), 3.87 (s, 3H, 7-OCH3), 1.54 (s, 3H, 4′-SCH3); Anal. calcd. for C17H16N2O3S: C, 62.18; H, 4.91; N, 8.53; O, 14.62; S, 9.76; found: C, 62.15; H, 4.93; N, 8.56; O, 14.64; S, 9.74.
**QNZ-5**	3-(4-butylphenyl)-6,7-dimethoxyquinazolin-4(3H)-one	Yield: 28.63%; Rf: 0.80 (ethyl acetate/hexanes, 7:3); 1H-NMR: (400 MHz, DMSO-d6): δH 8.21 (s, 1H, H-2), 7.49 (s, 1H, H-5), 7.40 (d, J2′,3′ = J6′,5′ = 8.4 Hz, 2H, H-2′, H-6′), 7.36 (d, J3′,2′ = J5′,6′ = 8.4 Hz, 2H, H-3′, H-5′), 7.19 (s, 1H, H-8), 3.92 (s, 3H, 6-OCH3), 3.87 (s, 3H, 7-OCH3), 2.67 (t, JCH2,CH2 = 7.6 Hz, 2H, 4′-CH2CH2CH2CH3), 1.63 (m, 2H, 4′-CH2CH2CH2CH3), 1.39 (m, 2H, 4′-CH2CH2CH2CH3), 2.67 (t, JCH3,CH2 = 7.2 Hz, 3H, 4′-CH2CH2CH2CH3); Anal. calcd. for C20H22N2O3: C, 70.99; H, 6.55; N, 8.28; O, 14.18; found: C, 70.97; H, 6.57; N, 8.24; O, 14.15.
**QNZ-6**	6,7-dimethoxy-3-(4-methoxyphenyl)quinazolin-4(3H)-one	Yield: 57.78%; Rf: 0.57 (ethyl acetate/hexanes, 7:3); 1H-NMR: (400 MHz, DMSO-d6): δH 8.18 (s, 1H, H-2), 7.48 (s, 1H, H-5), 7.42 (d, J2′,3′ = J6′,5′ = 8.8 Hz, 2H, H-2′, H-6′), 7.18 (s, 1H, H-8), 7.08 (d, J3′,2′ = J5′,6′ = 8.8 Hz, 2H, H-3′, H-5′), 3.92 (s, 3H, 6-OCH3), 3.87 (s, 3H, 7-OCH3), 3.81 (s, 3H, 4′-OCH3); Anal. calcd. for C17H16N2O4: C, 65.38; H, 5.16; N, 8.97; O, 20.49; found: C, 65.36; H, 5.13; N, 8.95; O, 20.44.
